# Complete Genome Sequence of Middle East Respiratory Syndrome Coronavirus KOR/KNIH/002_05_2015, Isolated in South Korea

**DOI:** 10.1128/genomeA.00787-15

**Published:** 2015-08-13

**Authors:** You-Jin Kim, Yong-Joon Cho, Dae-Won Kim, Jeong-Sun Yang, Hak Kim, SungHan Park, Young Woo Han, Mi-ran Yun, Han Saem Lee, A-Reum Kim, Deok Rim Heo, Joo Ae Kim, Su Jin Kim, Hee-Dong Jung, Namil Kim, Seok-Hwan Yoon, Jeong-Gu Nam, Hae Ji Kang, Hyang-Min Cheong, Joo-Shil Lee, Jongsik Chun, Sung Soon Kim

**Affiliations:** a Division of Respiratory Viruses, Center for Infectious Diseases, Korea National Institute of Health, Korea Centers for Disease Control and Prevention, Cheonju-si, South Korea; b ChunLab, Inc., Seoul National University, Seoul, South Korea; c Division of Biosafety Evaluation and Control, Korea National Institute of Health, Korea Centers for Disease Control and Prevention, Cheongju-si, South Korea; d Korea National Institute of Health, Korea Centers for Disease Control and Prevention, Cheongju-si, South Korea

## Abstract

The full genome sequence of a Middle East respiratory syndrome coronavirus (MERS-CoV) was identified from cultured and isolated in Vero cells. The viral genome sequence has high similarity to 53 human MERS-CoVs, ranging from 99.5% to 99.8% at the nucleotide level.

## GENOME ANNOUNCEMENT

Middle East respiratory syndrome coronavirus (MERS-CoV) is the first betacoronavirus lineage C member isolated from humans. It has been assumed that MERS-CoV was transmitted from bats and spread to humans through intermediate hosts (1). The genome structure is a single-stranded RNA (ssRNA) encoding 10 proteins; two replicase polyproteins (open reading frames [ORFs] 1ab and 1a), three structural proteins (E, N, and M), a surface (spike) glycoprotein (S), and five nonstructural proteins (ORFs 3, 4a, 4b, and 5) (2).

A sputum sample was collected from a second patient on 20 May 2015. The MERS-CoV was inoculated to Vero cells and passed three times. The RNA was isolated from the third viral culture solution with the QIAamp viral RNA mini kit (QIAGEN, Germany). Reverse transcription was performed with the Superscript III first-strand synthesis system (Life Technologies, the Netherlands) with specific-reverse primers. The cDNA was amplified by overlapping PCR primers based on a previous study (3). Additional PCR primers were designed for nonamplified regions. The resulting PCR amplicons were pooled and fragmented to an average 300-bp length, and the sequencing library was constructed with an Illumina TruSeq Nano DNA sample prep kit (Illumina, USA). The sequencing was performed with an Illumina MiSeq 50-bp single-end platform (Illumina). A total of 2,814,805 sequence reads were generated, and 2,617,936 reads (93.01%) were mapped to the consensus sequence from human-origin MERS-CoV genome sequences retrieved from GenBank. Mapping was accomplished by Bowtie version 2.2.4 (4) with default parameters. Finally, the whole viral genome sequence was obtained from the mapped result with an average coverage of 3,605.95×. Based on the assembly, the genome size was estimated to be 30,108 bp with a GC content of 41.15%.

The sequence analysis of South Korean MERS-CoV was performed with 53 complete genomes of human MERS-CoV available from GenBank using MUSCLE in the MEGA version 6 package (5). The full-genome sequence of MERS-CoV/KOR/KNIH/002_05_2015 showed overall nucleotide identities of 99.5% to 99.8% with 53 human MERS-CoVs. The overall identity to EMC/2012 (accession no. JX869059), the reference genome, was 99.5%. The closest strain was Hafr-Al-Batin_1 (accession no. KF600628) with 99.8% similarity.

In this analysis, the Korean MERS-CoV includes 29 nucleotides and 12 amino acid variants, compared to 53 full-genome sequences for human MERS-CoV. Two specific variations, Arg137Ser in the N-terminal domain and Leu530Val in the receptor-binding domain, whose spike proteins mediate virus entry and affect the viral host range, were identified only in the cell-cultured MERS-CoV/KOR/KNIH/002_05_2015 (compare with other variation studies of the receptor-binding domain of the spike protein [6]).

### Nucleotide sequence accession number.

The complete genome sequence of the MERS-CoV/KOR/KNIH/002_05_2015 isolate was deposited in GenBank under the accession number KT029139.
